# Witnessing the end, supporting the living: A qualitative study of palliative caregiving in end-of-life patients in Türkiye

**DOI:** 10.1017/S147895152610162X

**Published:** 2026-02-11

**Authors:** Selin Sert Yurdakul, Merve Erbay Erşen, Dilara Özel

**Affiliations:** 1 Department of Neuroscience, Bahcesehir University, Istanbul, Turkey; 2 Department of Clinical Psychology, Işık University, Istanbul, Turkey; 3 Department of Educational Sciences, Middle East Technical University, Ankara, Turkey

**Keywords:** Palliative care, palliative caregivers, anticipatory grief, caregiver resilience, body-oriented interventions

## Abstract

**Objectives:**

Palliative care seeks to enhance the quality of life for individuals with serious illnesses and their families by addressing physical, emotional, and psychological needs. This phenomenological study examines the lived experiences of 8 caregivers in palliative care settings in Türkiye, focusing on the challenges they face, the coping mechanisms they employ, and their reflections on the caregiving role. Special emphasis is given to both psychological and somatic signs of stress, along with the possible advantages of body-oriented resilience techniques.

**Methods:**

Using a phenomenological qualitative design, semi-structured interviews were conducted with 8 caregivers providing care to relatives in a hospital-based palliative care unit. Data were collected between February and April 2023 and analyzed through conventional content analysis.

**Results:**

Four central themes emerged from inductive coding: *harmony in healing, navigating difficulties, resilience in palliative care*, and *reflections on the finite*. The findings reveal a dual reality: palliative caregivers derive meaning and satisfaction from compassionate connections, high-quality clinical care, and peer support, yet they also endure significant burdens, including emotional strain, physical exhaustion, disrupted daily routines, and shifting relational dynamics. Anticipatory grief and chronic stress responses were prevalent, frequently manifesting in both psychological and somatic forms (e.g., sleep disturbances, muscle tension, and autonomic arousal). Despite these challenges, palliative caregivers employed spiritual beliefs, peer interactions, and self-care routines as resilience strategies.

**Significance of results:**

The mind–body challenges identified in the study emphasize the need for interventions that focus on self-regulation and resilience, including body-oriented approaches that strengthen internal resources, regulate stress responses, and encourage adaptability. Incorporating such approaches into group-based settings may improve mutual support and enhance both individual and relational well-being. The study highlights the importance of comprehensive, caregiver-centered support systems to reduce burden and improve the overall quality of palliative care.

## Introduction

Palliative care is a specialized, multidisciplinary approach designed to alleviate the physical, emotional, psychosocial, and spiritual distress associated with severe, life-limiting illnesses, irrespective of diagnosis or disease stage (Meier 2011; World Health Organization 2020). Its central aim is to enhance the quality of life and uphold the dignity of both patients and their families through comprehensive symptom management and holistic psychosocial support (Choo et al. 2019). Delivered by teams that typically include physicians, nurses, social workers, chaplains, and other specialists, palliative care not only addresses complex symptomatology but also assists individuals in navigating medical decisions and coping with the psychological and existential challenges of serious illness (Bakitas et al. 2009; Quill and Abernethy 2013; Teno and Clarridge et al. 2013). Despite providing the best possible care for patients and addressing caregivers’ well-being, additional measures are needed (O’Hara et al. 2010).

In Türkiye, palliative care has expanded through national initiatives such as the *Palliative Care Service Delivery Program* (MoH 2015), yet the system continues to emphasize biomedical management over psychosocial and family-centered care. Most services remain hospital-based rather than community-oriented, limiting relational continuity and heightening caregiver burden (Ersin and Bahar 2013). Despite institutional growth, care delivery still relies heavily on family members who perform nonclinical tasks such as feeding, hygiene, and emotional support (Göksel et al. 2020). This hybrid model reflects both cultural expectations and systemic gaps in staffing and psychosocial provision (Yardımcı and Mert 2024). Caregiving for seriously ill relatives is deeply embedded in Türkiye’s Islamic and collectivist traditions, where caring for a parent or spouse is viewed as a moral duty and spiritual act (Turgut and Soylu 2020; Yardimci and Mert 2024). The intersection of limited institutional resources, high emotional demands, and culturally reinforced caregiving norms creates an urgent need to understand palliative caregivers’ lived experiences, an area that remains underexplored.

Caregivers in palliative settings often face extensive physical, emotional, and psychological challenges, making it imperative to understand these experiences to improve support systems and palliative care services, as is the case in many other contexts across Türkiye as well (Turgut and Soylu 2020; Kutluk et al. 2021). Furthermore, as caregiving involves both psychological and embodied dimensions, attention to mind–body processes and their impact on palliative caregivers’ resilience has become increasingly relevant. The present study aims to explore caregivers’ lived experiences in palliative care settings in Türkiye, with particular attention to the challenges they face and the coping mechanisms they employ. Specifically, this study seeks to answer the following overarching research question: *How do caregivers in palliative care settings in Türkiye experience and make sense of the physical, emotional, and embodied aspects of caregiving?* By employing a phenomenological research design, this research seeks to provide a detailed and empathetic understanding of palliative caregivers’ perspectives, which can inform better support systems and care practices in palliative care units, and offer insight into the potential role of body-oriented approaches in fostering palliative caregivers’ resilience and overall well-being.

### Well-being of caregivers

The psychological well-being of palliative caregivers is a key determinant of their ability to provide compassionate and sustained care. Caring for terminally ill individuals entails persistent stress, anxiety, and depression driven by emotional demands and anticipatory grief, the mourning process that begins before death and continues afterward (Rando 1986; Hudson et al. 2004; Waldrop 2007; Holley 2009; Stajduhar et al. 2010; Bozdağ et al. 2025). This prolonged grief involves both preparation for loss and enduring a series of “mini-deaths,” marked by separation anxiety, sadness, guilt, and exhaustion (Marwit and Meuser 2002; Garand et al. 2012).

Relational disruptions such as changes in daily routines, loss of shared plans, and emotional distancing further intensify distress and highlight the need for timely psychosocial support (Williams and McCorkle 2011; Nielsen and Neergaard et al. 2017; Dutta et al. 2018; Axelsson et al. 2020). In dementia and other chronic illnesses, such grief responses may intertwine with unresolved issues from earlier stages of caregiving, reflecting Boss’s (1999) conceptualization of caregiving and grief as an ongoing relational process, where pre-death reactions such as sadness, anger, and longing coexist with gradual acceptance (Lindauer and Harvath 2014).

Beyond grief, caregivers face heightened risks of depressive and anxiety disorders, compounded by physical strain, social isolation, and financial hardship (Cameron et al. 2002; Grunfeld and Coyle et al. 2004; Kim et al. 2015). Effective coping strategies such as seeking social support, engaging in self-care, and fostering positive reappraisal can mitigate these effects and promote resilience (McPherson et al. 2007; Hudson and Payne 2011; Folkman 2013; Li et al. 2013).

### Enhancing caregiver resilience through body-oriented self-regulation strategies

Caregiver stress often manifests through autonomic dysregulation, muscle tension, hyperarousal, and sleep disturbance, revealing its deeply embodied nature (Grunfeld and Coyle et al. 2004). Body-oriented psychotherapies, which target autonomic regulation rather than cognitive reframing, seek to restore flexibility and resilience in the nervous system (Payne et al. 2015; Price and Hooven 2018; Kuhfuß et al. 2021) by engaging subcortical and sensory pathways that integrate bodily, emotional, and cognitive processes (Van der Kolk 2014; Corrigan and Hull 2015), mitigating long-term effects of chronic caregiving stress (Applebaum and Breitbart 2013). Within this broader class, approaches such as Somatic Experiencing (SE) (Levine 1997; Andersen and Lahav et al. 2017; Brom et al. 2017), Integral Somatic Psychology (ISP) (Niedenthal 2007; Selvam 2022), and the Bodynamic system (Marcher and Fich 2010) offer illustrative examples that emphasize bodily awareness and regulation; however, detailed mechanisms of these modalities are reserved for the discussion section, where they are interpreted alongside the study’s somatic findings.

Echoing findings from Özel (2024), body-oriented group therapy can strengthen interpersonal co-regulation, helping palliative caregivers feel seen, heard, and validated amid emotional isolation and role strain. Through shared exercises that build internal and external resources, participants learn via modeling, empathy, and universality, core therapeutic factors in group work (Yalom and Leszcz 2005). When professionally facilitated, such approaches foster emotional resilience and enhance caregivers’ well-being.

## Methodology

### Research design

This study employed a phenomenological research design to explore participants’ experiences in depth. Phenomenology aims to understand and describe how individuals perceive and make sense of their lived experiences (Moustakas 1994). This approach is particularly suited for investigating the complex and nuanced experiences of individuals involved in palliative care, as it allows for a detailed and empathetic exploration of their perspectives (Creswell and Poth 2018). In this study, semi-structured interviews were used to gather rich, descriptive data from participants.

### Participants

The study included 8 caregivers (5 females and 3 males), aged between 33 and 85 years, who were providing care to close family members, primarily spouses or parents, in palliative care settings. Most of the female caregivers were middle-aged, married, and unemployed, devoting their time solely to caregiving. The male participants varied in age and employment status; while one was employed and engaged in recreational activities such as camping and basketball, the others were retired or unemployed. Across the group, physical activity levels were generally low, and caregiving responsibilities significantly shaped their daily routines and lifestyles. In this paper, we use the term “palliative caregivers” to refer to family members providing bedside support in the palliative care unit, and “caregivers” as more broad term to align with international literature.

### Instrument

#### Semi-structured interview protocol

Researchers developed the semi-structured interview protocol to explore the experiences of individuals involved in palliative care. The protocol was designed with a focus on 4 key areas: a. demographic information, b. palliative care experiences, c. emotional and psychological impacts, future outlook, and d. coping mechanisms. To explore participants’ experiences, the interview includes open-ended questions like, “*Can you talk about your experiences in palliative care?*” and “*What are the positive aspects of palliative care?*” This approach enables participants to share their personal stories and insights, providing a comprehensive understanding of their experiences. The semi-structured interview protocol allows for an extensive exploration of participants’ experiences while providing the flexibility to probe deeper into specific areas as needed.

### Research procedure

Data were collected between February and April 2023 after obtaining ethical approval from the Middle East Technical University (METU) Human Subjects Ethics Committee. Pilot interviews were first conducted to refine the semi-structured guide and ensure question clarity and relevance. Participants were recruited through convenience sampling via the professional network of a hospital-based researcher, allowing efficient access to individuals actively engaged in palliative caregiving. Each semi-structured interview lasted approximately 90 min. Participants received information on the study’s purpose, voluntary participation, confidentiality, and expected duration, and all provided written consent for audio recording. This process ensured ethical compliance, participant safety, and the collection of rich, credible narratives reflecting the lived experiences of palliative caregivers.

### Data analysis

Interview data were analyzed through conventional qualitative content analysis, enabling inductive theme generation (Hsieh and Shannon 2005). Verbatim transcripts ensured accuracy (Braun and Clarke 2006). Researchers independently conducted repeated readings and open coding, organizing data into categories and overarching themes (Elo and Kyngäs 2008). To strengthen credibility, 2 independent coders with no connection to the palliative unit analyzed the data, and all 3 researchers held dialogical meetings to reconcile differences and ensure reflexive rigor (Patton 2015). Verbatim quotations were included to enhance transparency and transferability (Lincoln and Guba 1985). Participant identifiers (“CG1,” “CG2,” etc.) preserve confidentiality while distinguishing individual voices.

### Researcher and language reflexivity

#### Researcher reflexivity

As the physician overseeing this study and the first author, I had prior clinical relationships with several participants, which shaped both access and interpretation. Conducting interviews outside the clinical setting allowed a deeper understanding of their emotional and caregiving realities and fostered mutual empathy. This connection also illuminated how our relationship itself supported caregivers’ resilience, an insight reflected in the study’s themes. To address the influence of my dual role, procedural and reflexive strategies were used to ensure rigor. All interviews took place in neutral, nonclinical settings, with participants’ autonomy and confidentiality emphasized. Continuous reflexive journaling and an audit trail documented interpretive decisions and potential bias, transforming positionality from a limitation into an epistemic strength.

#### Language reflexivity

Drawing on the arguments of Hamid and Oyinloye (2025), we chose to preserve participants’ quotations in their original form as much as possible. Since the interviews were conducted in Turkish, certain discrepancies may exist between spoken and written language, and traces of everyday expressions are therefore retained in the excerpts. During translation into English, particular attention was given to lexical choices that might convey hierarchical nuances. Rather than modifying the text to make it linguistically polished, we intentionally maintained the authenticity and texture of participants’ voices to honor their linguistic and cultural expressions.

## Results

**Figure 1.**
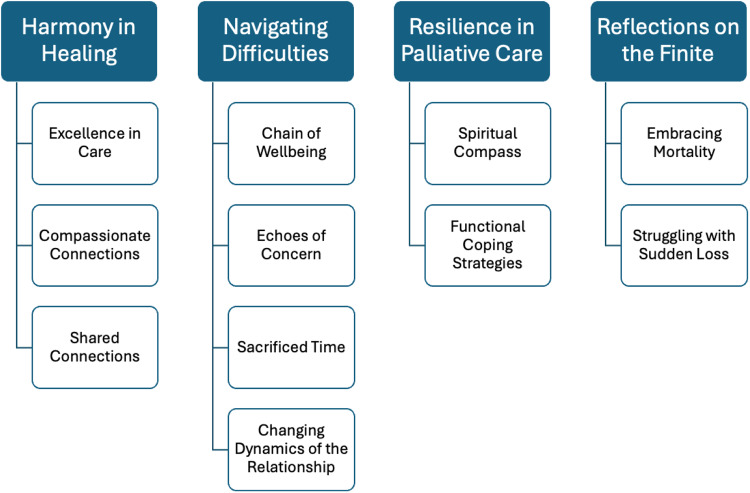
Results of the needs analysis.

The qualitative analysis of interviews with relatives of patients receiving palliative care identified 4 overarching themes: *harmony in healing, navigating difficulties, resilience in palliative care*, and *reflections on the finite*. The detailed structure of these themes, along with their corresponding subthemes, is presented in Figure 1.

### Harmony in healing

Within the positive experiences reported by participants, 3 core codes were identified: *excellence in care, compassionate connections*, and *shared connections*. The first code, *excellence in care*, refers to the perceived high quality of clinical and operational services provided to patients. A substantial proportion of participants (75%) expressed satisfaction with the standard of care their relatives received. These accounts highlight the exceptional quality, cleanliness, and comfort available within the palliative care unit. One participant, whose relative had been in the facility for 5.5 months, stated:

*We are especially pleased with our doctor, and the staff is good. The treatments are very nice, and I can say it’s more comfortable than our home. We feel comfortable here, and the care and cleanliness are top-notch* (CG3).

The second code, *compassionate connections*, highlights the importance of respectful and empathetic relationships with healthcare providers. The interviews illustrate that interpersonal relationships and social support within the palliative care unit significantly enhance caregivers’ experiences. One participant described:

*Personal relations are good, and so are the colleagues. We are mostly in contact with the nurses. I have never seen them being disrespectful. They understand the patient’s condition and try their best to help. That’s the most positive aspect. They take care of my father as if he were their own, talking and chatting with him* (CG1).

Another participant reflected on the unexpected strength of the relationships formed:

*I thought this place would be like a regular ward, but it turned out to be a place where I formed stronger bonds, with doctors, nurses, and other caregivers* (CG2).

Similarly, a third participant emphasized the familial atmosphere and ease of interaction:

*It has been progressing very poorly for 2 years, but time passed more quickly after coming here. I can sit with the staff like a family. We knock on your door and ask questions, or call you on the phone. I am very satisfied* (CG3).

These accounts reveal the central role of compassionate engagement in fostering trust, emotional safety, and a sense of belonging within the care environment.

The third code, *shared connections*, reflects the supportive relationships formed among caregivers of different patients. Participants emphasized the value of shared understanding and solidarity. One participant noted: “*This place feels like the final stop for patients, so we share the same situation and pain with other patient relatives, which creates a bond. We also form a bond with our doctors*” (CG2). Another participant described this camaraderie as a mutual coping resource: “*Inside the hospital, we become like partners in distress with other caregivers. If there are any issues, we share and talk about them*” (CG1). These reflections underscore the psychosocial importance of peer connections in alleviating isolation, facilitating emotional expression, and promoting resilience among caregivers in palliative care settings.

### Navigating difficulties

The second theme, *navigating difficulties*, encompasses 4 interrelated codes: *chain of wellbeing, echoes of concern, sacrificed time*, and *changing dynamics of relationships*. The first code, *chain of wellbeing*, captures the significant physical, psychological, and social strain experienced by all participants. Commonly reported challenges included fatigue, sleep disturbances, reduced concentration, depressive symptoms, and neglect of self-care. As 1 participant explained, “*Sometimes I feel like withdrawing from life completely. When the moaning starts, I can’t find any time for myself*” (CG2). Another noted, “*It’s very difficult, but when it’s someone you love, you don’t even notice. You forget yourself*” (CG3). These accounts illustrate the cumulative toll caregiving exacts on both mental and physical health.

The second code, *echoes of concern*, reflects the distress associated with patients’ worsening conditions, persistent symptoms, and the fear of impending loss. Caregivers described feelings of helplessness in the face of pain, physical decline, and behavioral changes, often accompanied by heightened anxiety and anticipatory grief. One participant shared, “*She is wasting away before my eyes, and I can’t do anything*” (CG3), while another expressed, “*It feels like time is running out, and we are going to lose him*” (CG4).

The third code, *sacrificed time*, highlights the disruption of daily routines, work, and social life. Many participants reported prioritizing caregiving responsibilities to the extent that personal, professional, and social engagements were significantly curtailed. One remarked, “*I have a house, 2 children, and a job… After 55 days, it’s just too much*” (CG1). Another reflected, “*When I come here, I inevitably lose touch with my friends*” (CG5).

The fourth code, *changing dynamics of relationships*, addresses the role reversals and emotional adjustments necessitated by the progression of illness. Caregivers often shifted from perceiving their loved one as “strong” to adapting to their more fragile and dependent state. As 1 participant described, “*I’ve become my father’s father. There’s nothing else to do*” (CG1). These experiences underscore the profound emotional recalibration required in the caregiving role.

Collectively, these 4 codes illustrate the multidimensional challenges caregivers face in palliative care, underscoring the need for integrated support systems that address physical health, emotional resilience, social connection, and relational adjustment.

### Resilience in palliative care

This theme comprises 2 codes: *spiritual compass* and *functional coping strategies*. Under *spiritual compass*, most participants (88%) framed caregiving as a morally meaningful and fulfilling act, describing it as a duty, an expression of love, and a source of personal peace. As 1 caregiver stated, “*I see it as a good thing. I believe I’ve done my best. My conscience is clear*” (CG3). Others emphasized reciprocity: “*Our father raised us and brought us to this age. It is our duty to take care of him*” (CG8). Such perspectives provided emotional grounding, helping caregivers navigate the challenges of palliative care.

Participants identified *functional coping strategies* to sustain well-being, including social support, physical activity, and self-care. One caregiver described, “*I talk to the nurses; I have friends I call. I share my troubles, cry, and get support*” (CG3). Others mentioned taking walks, meeting friends, and engaging in activities that fostered personal renewal: “I try to take time for myself, go to the hairdresser, buy clothes, and make myself happy” (CG2). These strategies highlight the role of both relational and self-focused practices in maintaining resilience.

### Reflections on the finite

This theme includes *embracing mortality* and *struggling with sudden loss*. In *embracing mortality*, participants described how frequent exposure to death in the palliative care unit reshaped their perspectives on life’s fragility. As one noted, “*Death is like a big film in the middle of the room, but everyone pretends not to see it… I have become friends with it*” (CG1). Another reflected, “*I’ve witnessed more than 20 deaths here… Staying in palliative care means facing death*” (CG2). These encounters fostered acceptance of mortality and a heightened appreciation for health and life’s impermanence.

In *struggling with sudden loss*, caregivers recounted the shock of losing a loved one unexpectedly, often within months of apparent health. “*Losing my father, someone so close, is very different… I wasn’t ready at all*” (CG4). Others described the difficulty of maintaining emotional composure for dependents: “*It’s very hard, extremely hard, but I have to hold myself together for them*” (CG7). Fear of the patient’s death and its practical implications also weighed heavily, underscoring the emotional and existential toll of caregiving in palliative settings.

## Discussion

This study provides a nuanced, layered understanding of caregiving in palliative care, illuminating both its deeply emotional rewards and its profound burdens. The first core theme highlights palliative caregivers’ appreciation for compassionate, coordinated healthcare, especially providers’ empathetic communication and relational presence. This aligns with established findings that caregiver distress is mitigated when healthcare teams promote transparent dialogue and relational attunement (Lall et al. 2020; Engel et al. 2023). Peer connections also played a critical role; co-caregivers provided not only emotional solidarity but also a shared understanding and recognition that professional relationships alone cannot fulfill (Hudson et al. 2004; Engel et al. 2023; Shimizu et al. 2025).

Yet, caregiving was also characterized by emotional upheaval: anticipatory grief, fluctuating identity, and the agony of wishing for both the end of suffering and the continued presence of loved ones. These dualities reflect the ambivalent nature of pre-loss grief described in prior palliative studies (Hudson and Payne 2011; Coelho et al. 2020). Emergent themes underscored how social withdrawal, shifting family roles, and existential uncertainty intensify psychosocial distress. This mirrors prior work demonstrating that disrupted relational boundaries and role transitions significantly contribute to caregiver vulnerability (Best and Aldridge et al. 2015; Nielsen and Neergaard et al. 2017).

The third theme in this study relates to the coping strategies that palliative caregivers use to manage their complex challenges. Participants described a range of strategies, including the use of spiritual beliefs, engagement in self-care practices, and active solicitation of social support, as essential for sustaining resilience echoing previous research (Folkman 2013; Li et al. 2013). Our findings resonate with previous research highlighting spirituality and existential meaning-making as vital mechanisms for endurance among palliative caregivers (Paiva et al. 2015; Arian et al. 2017; Gijsberts et al. 2019). Palliative caregivers’ reliance on spiritual practices and ritual mirrored earlier evidence that such meaning-making fosters coherence, hope, and relational connection in the face of loss (Li et al. 2013; Best et al. 2023).

Besides the emotional and relational challenges frequently noted in caregiving, our findings revealed a clear somatic dimension of distress, characterized by persistent muscle tension, disrupted sleep, and autonomic overstimulation. These embodied manifestations highlight grief and caregiver strain as not only psychological but deeply physiological experiences. Accordingly, interventions must address both the emotional and bodily sequelae of caregiving. Body-oriented approaches that enhance nervous system regulation and resilience – such as resource-building and interoceptive tracking – can help caregivers tolerate dysregulation without becoming overwhelmed (Levine 1997, 2010; Payne et al. 2015; Kuhfuß et al. 2021; Selvam 2022). By restoring autonomic balance and coherence between bodily and emotional states, such methods offer promising avenues for fostering sustained well-being among palliative caregivers.

Caregivers’ descriptions of chronic muscular bracing, difficulty down-regulating after distress, and a sense of “carrying tension in the body” parallel SE’s account of incomplete defensive responses that persist as autonomic activation (Levine 1997, 2010), and their oscillation between numbness and overwhelm reflects SE concepts of constriction and sympathetic flooding, indicating that titrated pendulation may be particularly relevant. Similarly, ISP’s focus on increasing affect tolerance through expanding the body’s capacity to contain emotional states (Selvam 2022) aligns with caregivers’ experiences of emotional destabilization when bodily sensations intensified, highlighting the value of strengthening interoceptive awareness to detect early autonomic shifts. The Bodynamic model further illuminates caregivers’ reports of tension in the shoulders, chest, and diaphragm, which are the areas associated with relational boundaries and responsibility, consistent with its mapping of muscle groups to functions such as caretaking, vigilance, and self-protection (Marcher and Fich 2010). Together, these frameworks suggest that chronic caregiving responsibilities may become embodied through patterns of overactivation and collapse across musculoskeletal and autonomic systems.

Importantly, palliative caregivers described marked somatic and affective dysregulation, persistent muscle tension, sleep disturbance, and autonomic overstimulation, which highlight grief as an embodied phenomenon. This underscores the therapeutic need to address physical as well as psychological sequelae. Integrating somatic mechanisms into intervention design such as titrated discharge of physiological activation, expansion of affective holding capacity, and recalibration of muscular patterns may be particularly beneficial for caregivers experiencing chronic stress. Body-oriented interventions, which may directly target this embodied distress and enhance self-regulation and resilience, might help palliative caregivers regulate their nervous systems, titrated discharge of physiological activation, and re-establishing coherence between emotional and bodily states (Levine 1997; Payne et al. 2015; Selvam 2022). Techniques like resource-building and interoceptive tracking may help them to tolerate dysregulation without being overwhelmed (Levine 2010; Kuhfuß et al. 2021).

Given the emotional and relational demands of palliative caregiving, group-based body-oriented interventions offer a congruent therapeutic framework. Through practices such as pendulation, titration, and interoceptive tracking, these approaches foster somatic regulation while cultivating interpersonal safety and validation (Özel 2024). Within this collective setting, caregivers develop embodied self-regulation skills and experience the reparative effects of co-regulation, critical buffers against isolation, emotional overload, and burnout. The group context further supports identity integration and mutual recognition, mitigating role confusion and existential strain (Yalom and Leszcz 2005). Thus, body-oriented group interventions extend beyond symptom relief, functioning as relational ecosystems that strengthen resilience and restore caregivers’ capacity for connection and meaning-making.

### Limitations

This study has several methodological and contextual limitations. Conducted in a single hospital with a relatively small sample size, its findings should be interpreted with caution and may not be generalizable to broader caregiver populations or other institutional contexts. The dual role of 1 researcher as the lead physician in the palliative care unit may have influenced participants’ willingness to speak freely, potentially introducing response bias. To address this concern, several reflexive methodologies were employed and explained in the reflexivity section to enhance transparency and analytic rigor. Additionally, as participation was voluntary, self-selection bias may have occurred, possibly overrepresenting caregivers who were more reflective or communicative about their experiences.

Cultural context further shapes how these findings should be interpreted. In Türkiye, caregiving is closely tied to collectivist family values and moral expectations emphasizing duty and self-sacrifice, while faith-based coping, particularly Islam’s emphasis on patience, provides caregivers with moral grounding and emotional endurance (Sunar and Fişek 2005; Kağıtçıbaşı 2007; Turgut and Soylu 2020; Kutluk et al. 2021). At the same time, these norms may discourage the open expression of distress or help-seeking due to stigma or perceived moral obligations (Ersin and Bahar 2013; Özdemir et al. 2021). Given that Muslim and collectivist populations are globally dispersed, these insights hold broader relevance for multicultural caregiving contexts, underscoring the importance of culturally attuned and transferable support frameworks.

### Clinical implications

This study underscores the clinical relevance of integrating mind–body-oriented interventions into palliative care to address caregivers’ embodied stress responses and emotional exhaustion. Future research should evaluate somatic and body-oriented psychotherapies across diverse caregiving populations, using longitudinal and controlled designs to assess their long-term effects on well-being and relational functioning.

In clinical translation, such interventions could be delivered by psychotherapists, clinical psychologists, or palliative care counselors with additional certification in somatic or trauma-informed modalities. Referrals may be particularly appropriate for palliative caregivers exhibiting chronic tension, compassion fatigue, emotional dysregulation, or somatization. Implementation within existing care structures, through small-group or dyadic formats integrated with psychoeducation and support groups, can enhance bodily awareness, emotion regulation, and presence. Hybrid delivery formats combining in-person and online sessions may further increase accessibility and continuity of care.

At the systemic level, cross-disciplinary collaboration among trauma specialists, palliative care teams, and palliative caregiver organizations will be essential for embedding mind–body interventions into standard care pathways. Future pilot protocols adapted to the cultural and psychosocial stressors of caregiving should be tested for feasibility, safety, and scalability through randomized controlled trials. Ultimately, integrating body-oriented approaches within palliative frameworks can complement existing psychosocial models by addressing the somatic dimension of palliative caregiver stress, thereby enhancing resilience and improving the overall quality of palliative care.

## Conclusion

This study underscores the emotional and relational complexity of caregiving in palliative contexts, revealing a shared human tension between connection and anticipatory loss. While cultural factors shape expressions of care, the underlying experiences of grief, love, fatigue, and meaning-making transcend socio-cultural boundaries. The findings affirm that palliative caregivers are not only care providers but also invisible patients whose psychological and somatic burdens require deliberate attention. Integrating somatically informed, group-based support into palliative care may offer a pragmatic, evidence-informed strategy to address embodied stress, reduce isolation, and restore affective resilience. Such interventions hold promise not only for improving caregiver well-being but for reinforcing the relational integrity that lies at the heart of compassionate, high-quality palliative care.
